# Development and independent validation of explainable radiomics-based machine learning models for prognosis in colorectal liver metastases

**DOI:** 10.3389/fdgth.2025.1752699

**Published:** 2026-01-19

**Authors:** A. Brunetti, G. M. Zaccaria, E. Sibilano, S. Marzi, A. Vidiri, V. Bevilacqua

**Affiliations:** 1 Department of Electrical and Information Engineering, Polytechnic University of Bari, Bari, Italy; 2 Medical Physics Laboratory, IRCCS Regina Elena National Cancer Institute, Rome, Italy; 3 Radiology and Diagnostic Imaging Department, IRCCS Regina Elena National Cancer Institute, Rome, Italy

**Keywords:** CRLM, explainable machine learning, feature interpretability, prognosis, radiomics

## Abstract

**Introduction:**

Colorectal cancer frequently leads to liver metastases (CRLM), posing a major challenge to long-term survival. Prognosis remains heterogeneous, and traditional clinical risk scores often lack biological depth and spatial information. Advances in radiomics and machine learning (ML) offer the potential for improved, explainable outcome prediction; however, robust and interpretable prognostic models for CRLM remain an unmet need. This study aimed to develop and validate explainable ML models based on radiomic features extracted from both metastatic lesions and background liver tissue, enhancing the prediction of recurrence and overall survival (OS) status in patients with CRLM.

**Materials and methods:**

Patient data and contrast-enhanced CT images from two independent cohorts were analysed: a publicly available TCIA-CRLM series, employed as the discovery set, and a real-life clinical cohort, used as an external validation set. Segmentation focused on the largest liver metastasis (L-MAX) and surrounding healthy liver tissue (L-BKG), extracting radiomic features from both areas and their ratios (L-MAX/L-BKG). An end-to-end pipeline for data preprocessing and classification was designed. Multiple ML and Deep Learning (DL) classifiers were trained and validated. Model interpretability was assessed using SHapley Additive exPlanations (SHAP) analysis to identify key predictive radiomic determinants. Performances were compared to recognized clinical models.

**Results:**

For recurrence prediction, the best-performing classifier was a soft-voting ensemble of a multilayer perceptron (MLP) optimized via a Genetic Algorithm (GA); for OS status classification, the best performance was obtained by a hard-voting ensemble of a GA-optimized MLP. Both classifiers demonstrated robust discrimination capabilities in external validation, with AUCs of 0.78 and 0.68, respectively. The explainability analysis performed with SHAP revealed the most relevant radiomic determinants in the classification. These features retained prognostic significance in the independent cohort, supporting their use for clinical risk stratification.

**Discussion:**

Explainable ML models leveraging both lesion-centric and contextual liver radiomics offer clinically transparent prediction of recurrence and survival in CRLM. SHAP highlighted clinically plausible, reproducible imaging determinants, enabling risk stratification. The validation of specific radiomic determinants suggests the potential practical utility of this approach, laying out the groundwork for integrating with DL and multi-omic data in future oncology strategies.

## 1 Introduction

Colorectal cancer (CRC) is a leading cause of cancer-related mortality globally (1, 2), and the liver is the most common site of metastasis in CRC, primarily due to portal venous (PV) drainage from the bowel (3, 4). Colorectal liver metastases (CRLM) occur in 25%–50% of patients during the disease course; around 15%–25% present synchronously at diagnosis, with a further 18%–25% occurring metachronously within five years (3, 5–7). Prognosis is heterogeneous and influenced by clinical and pathological factors. Despite advances in surgery and systemic therapy, predicting overall survival (OS) and disease-free survival (DFS) remains challenging (8–10). Traditional prognostic models, such as the Fong clinical risk score, rely on clinical variables and basic imaging features but may fail to capture the underlying tumor biology and spatial heterogeneity that advanced image-based methods can address (11–14).

Radiomics enables the high-throughput extraction of quantitative imaging features that describe tumor shape, texture, and intensity patterns, providing a non-invasive method for characterizing tumor phenotype (15). When combined with Machine Learning (ML), radiomic data may overcome conventional statistical approaches, showing promise in enhancing the prognosis in cancer patients (16–18). Nevertheless, the increasing complexity of ML models has introduced the challenge of ensuring clinical interpretability, since many clinicians are reluctant to use “black box” models with opaque decision-making processes. Consequently, there is a need for ML-based prognostic approaches and tools that combine high performance with transparency regarding their decision logic (19).

Although radiomics in CRLM has been widely studied (13, 20), only a few works have investigated radiomics-based approaches combined with ML models, reporting promising but incomplete advances; even fewer studies focused on improving the explainability of these models. Fu et al. and Mühlberg et al. employed CT-based models to predict survival endpoints but encountered limitations in interpretability, which can be attributed either to the feature selection based on statistical methods or the lack of external validation (21, 22). Granata et al. applied K-Nearest Neighbor (K-NN) models to CT and MRI radiomics for liver recurrence prediction, yet their work lacked transparent explanations (23, 24). Simpson et al. used texture analysis to stratify hepatic recurrence risk but did not integrate ML approaches (25). Saber et al. explored explainability using TabNet and SHAP (SHapley Additive exPlanations), although their models were not independently validated (26). Overall, the development of robust and interpretable classification approaches remains an unmet need.

This work aims to develop and validate explainable pipelines for predicting prognostic outcomes, i.e., recurrence and OS status, in patients with CRLM. Specifically, radiomic features were extracted not only from metastatic lesions, but also from the surrounding liver parenchyma, which has often been overlooked in prior research, although evidence suggests it can reflect systemic or local effects of the disease and contain prognostic information complementary to a lesion-based analysis (27). Features were obtained from the largest lesion (L-MAX) and background liver tissue (L-BKG); also, feature ratios (L-MAX/L-BKG) were computed to integrate contextual information into predictive models.

To improve existing approaches, several pipelines based on ML and Deep Learning (DL) classifiers were developed and benchmarked. Models were trained on a publicly available dataset and externally validated on a real-life independent clinical cohort. Then, SHAP was employed to enhance interpretability, providing game-theoretic explanations of individual feature contributions to predictions, thus enabling the identification of the most influential radiomic features for prognosis and elucidating their interactions.

## 2 Materials and methods

### 2.1 Data collection

Individual data from newly diagnosed CRLM patients were collected from The Cancer Imaging Archive (TCIA)-CRLM series (25, 28), which includes 197 patients affected by CRLM, and a real-life cohort of 66 patients with at least one measurable CRLM as defined by the RECIST 1.1 Criteria (greater diameter ≥ 5 mm), which were evaluated from September 2012 to January 2021 at the National Cancer Institute Regina Elena (IRE-IRCCS, Rome, Italy) (29). For the IRE-IRCCS, the study was approved by ethics committees or institutional review boards (n. RS1765/22) and was conducted in accordance with the Declaration of Helsinki. Given the retrospective nature of the study, informed consent was waived.

The TCIA-CRLM dataset included contrast-enhanced CT image series acquired during the PV phase. A total of 197 CT series were labelled, and liver, tumors, and vessels were semi-automatically segmented as reported in Simpson et al. (25), which also provides all the details of the CT acquisition protocols. CT images were downloaded in DICOM format, and the volumes of interest (VOIs) were in DICOM Segmentation Object format. For the IRE-IRCCS series, all enrolled patients underwent a primary staging CT, with 66 image series acquired. The CT acquisition was performed using a variety of multidetector-row CT scanners from different manufacturers (Philips, Brilliance 64, Philips Medical Systems, Best, The Netherlands; Siemens Somatom Definition Flash, Siemens Healthcare, Erlangen, Germany; GE Lightspeed VCT, GE Medical Systems, Waukesha, WI, USA). The PV phase was obtained with a tube voltage of 120 kV and with a tube current covering 300–500 mA, with a scan delay set at 70 s. A weight-adapted contrast medium was administered intravenously at a rate of 2.0–3.0 mL/s, followed by a 40 mL saline flush. Images were acquired with a large field of view, a slice thickness range from 2.0 to 5.0 mm, and a pitch of 0.5–0.7; subsequently, a reconstruction algorithm was applied with a standard soft tissue kernel. The VOIs used in the subsequent radiomic analysis were delineated using the Pinnacle3 treatment planning system (version 16.2.1, Philips Healthcare Nederland B.V.). This software, which operates on Solaris UNIX (or UNIX-compliant) workstations, includes various modules for radiation therapy planning, including a contouring tool for defining targets and organs at risk. In cases with up to five visible metastases, both the entire liver and the adjacent, tumor-free parenchymal tissue were delineated separately on the PVP of the primary staging CT scan. A radiologist manually contoured each lesion using the paintbrush tool, while the liver was segmented using Pinnacle's Auto-Segmentation tool. This tool applies probability-based atlases and a segmentation algorithm to generate liver contours, which were then manually refined by the radiologist as needed.

Consistent with the TCIA-CRLM series, a subset of 34 patients from IRE-IRCCS was selected, excluding those with less than 24 months of follow-up for OS. For each patient, in both cohorts, recurrence and OS status were annotated. In particular, the follow-up time was calculated from the date of liver surgery to the date of the last follow-up or death. At the last follow-up, each patient was recorded as alive or alive with disease; the event of death was recorded as death due to disease or death due to other causes. Furthermore, the time of recurrence (in months) was calculated from the date of CRLM surgery (or the date of the bowel surgery in the case of synchronous colon and liver surgery) to the date of the first recurrence/local recurrence. A recurrence was defined as the identification of a new lesion on follow-up CT scans after liver resection, whereas local recurrence indicated an intrahepatic recurrence following the initial liver resection.

### 2.2 Workflow

**Figure 1:**
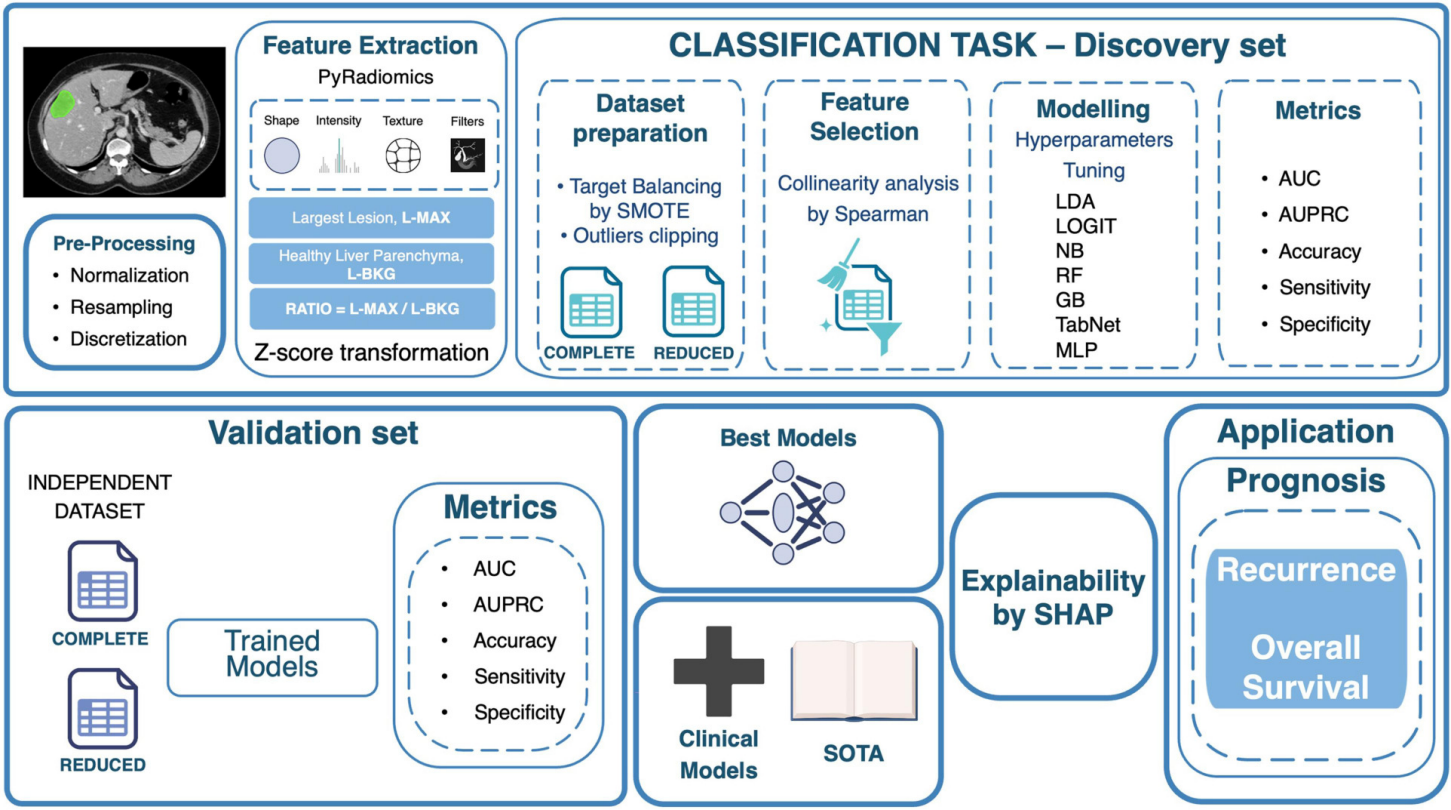
Workflow of the proposed radiomics-based classification framework. The pipeline includes image pre-processing, feature extraction (largest lesion, healthy liver parenchyma, and their ratio), feature selection, dataset preparation, model training with hyperparameter optimization, and performance evaluation on discovery and validation sets. The best models undergo explainability analysis with SHAP, and selected features are applied for prognostic evaluation of recurrence and overall survival. L-MAX: Largest Lesion; L-BKG: Liver Background; SMOTE: Synthetic Minority Over-sampling Technique; LDA: Linear Discriminant Analysis; LOGIT: Logistic Regression; NB: Naive Bayes; RF: Random Forest; GB: Gradient Boosting; MLP: Multi-Layer Perceptron; AUC: Area Under the Receiver Operating Characteristic curve; AUPRC: Area Under the Precision-Recall Curve; SOTA: State of the Art. Icons created using BioRender (https://www.biorender.com/), licensed under Academic License.

**Figure 2:**
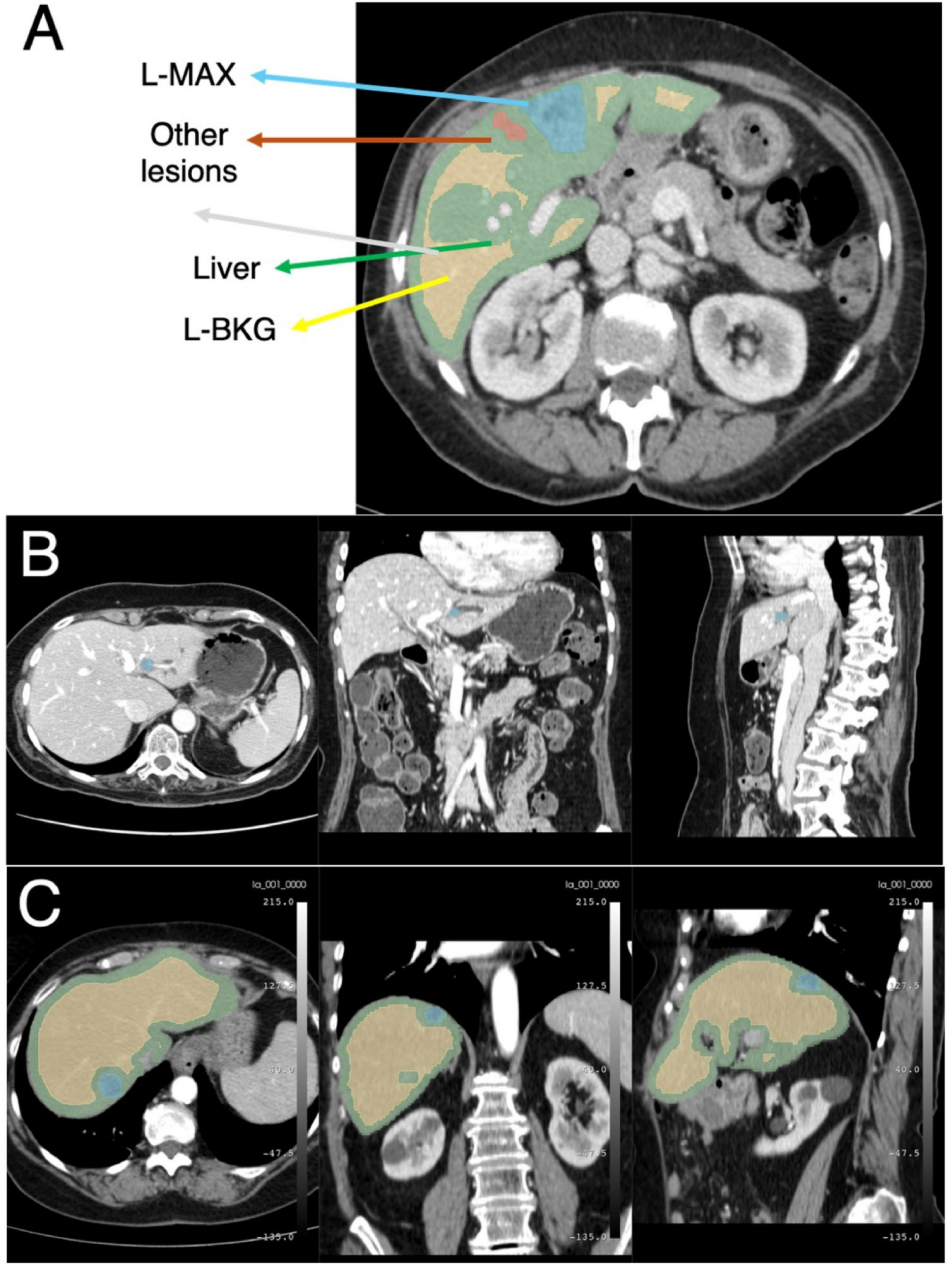
Example of CT images. **(A)** Example of CT-based segmentation highlighting the largest lesion (L-MAX) and smaller lesions, healthy liver parenchyma (L-BKG), and surrounding anatomical structures. Axial, coronal, and sagittal views from the discovery set **(B)** and from the validation set **(C)**.

Figure 1 summarizes the proposed end-to-end workflow. After image preprocessing, radiomic features were extracted from two volumes of interest, i.e., the L-MAX and the L-BKG, for both the TCIA-CRLM and the IRE-IRCCS image series. For each cohort, two sets of features were created: (i) the L-MAX features set; (ii) the RATIO features set, obtained from the element-wise ratio between the L-MAX and the L-BKG features. Figure 2A depicts an example of CT-based segmentation of liver lesions and parenchyma. L-MAX (blue) indicates the biggest lesions; L-BKG (green) represents parenchyma, with a 5 mm border from any lesions within the liver and from the liver surface (yellow). Figures 2B,C show axial, coronal, and sagittal views of a series from the discovery and the validation set, respectively.

The classification pipeline was then developed on the TCIA-CRLM image series dataset. This pipeline includes preliminary dataset preparation and feature selection, followed by the design, optimization, and evaluation of classification models.

Trained models were tested on the IRE-IRCCS image series dataset; subsequently, an explainability analysis was performed, enabling the identification of the most important radiomic determinants in the classification outcome.

### 2.3 Image preprocessing and radiomic feature extraction

Image preprocessing was performed using the PyRadiomics framework (version 3.1.0, Python version 3.8), which consisted of image resampling, intensity normalization, gray-level discretization, and radiomic feature extraction (30). PyRadiomics implements methods for extracting radiomic features in compliance with recognized standards (31). Intensities were normalized to a fixed scale of 500, voxel spacing was resampled to [1, 1, 1] mm using B-spline interpolation, and discretization was applied with a fixed bin width of 25, which has shown good reproducibility and performance in previous works.

Agnostic 3D features extracted from VOIs were considered, and L-MAX and RATIO datasets were generated (27, 32). Feature extraction was yielded from the original, the Laplacian of Gaussian (LoG) filtered, and the wavelet-transformed VOIs. The sigma parameter for the LoG filter varied from 1 to 5, with ticks spaced by 1 mm each. For the wavelet-transformed images, eight decompositions were obtained with Coiflets from 3D volumes.

First- and second-order (textural) features were extracted. Textural-based features can be derived from the Gray Level Co-occurrence Matrix (GLCM) (33, 34), the Gray Level Run Length Matrix (GLRLM) (35), the Gray Level Size Zone Matrix (GLSZM) (36), the Gray Level Dependence Matrix (GLDM) (37), or the Neighboring Gray Tone Difference Matrix (NGTDM) (38). For both L-MAX and the RATIO feature sets of computation, we assumed the TCIA-CRLM as the discovery set and the IRE-IRCCS dataset as the independent validation set. Thus, we preprocessed all datasets by applying a *Z*-score transformation of radiomic values. To avoid data leakage during analysis, the mean and standard deviation from the discovery set were applied when processing the validation set. This ensures that only information from the discovery set guides the validation, preventing unintentional sharing of data patterns between sets.

### 2.4 Classification

#### 2.4.1 Datasets preparation

To address class imbalance in the discovery set in reference to both the analyzed clinical endpoints, i.e., the recurrence and the OS status, the SMOTE algorithm (39) was employed from the imbalanced-learn library (version 0.13.0). Synthetic samples of the minority class were generated via interpolation. For each radiomic feature, differences in distribution between the discovery and validation sets were addressed by clipping outliers at the 0.05 and 0.95 quantiles. The boundaries estimated from the discovery set were subsequently applied to the validation set consistently, as for the previous *Z*-score transformation.

For both datasets, dimensionality reduction was performed with Spearman correlation analysis. Features with pairwise correlation coefficients below 0.5 were retained to minimize the loss of potentially relevant information. This analysis was conducted in R using the corrplot package (version 0.95). For the subsequent steps, both the full and the reduced datasets were considered.

#### 2.4.2 Classification models and performance assessment

Seven classification models were employed and compared: Linear Discriminant Analysis (LDA), Logistic Regression (Logit), Naive Bayes (NB), Random Forest (RF), Gradient Boosting (GB), TabNet, and MLP. LDA, Logit, NB, RF, and GB were implemented using the scikit-learn (version 1.2.2), MLP was implemented using both scikit-learn and TensorFlow frameworks (version 2.12.0), and TabNet was implemented via the PyTorch TabNet package (v3.1.1). Models' performances were evaluated using a stratified 5-fold cross-validation approach, with stratification based on the outcome.

Table 1 summarizes the optimization strategies adopted for the hyperparameters of the classifiers. For the traditional ML models, hyperparameters were optimized using a 5-fold stratified *RandomizedSearchCV*, with the objective of maximizing ROC-AUC on the internal validation set; the resulting best parameters were then fixed via *set_params* before training the final model and performing external validation.

**Table 1:** Summary of hyperparameter optimization strategies and search spaces for each classifier. SVD: Singular Value Decomposition; LSQR: Least Squares QR; Logit: Logistic Regression; LBFGS: Limited-memory Broyden–Fletcher–Goldfarb–Shanno; SAGA: Stochastic Average Gradient Augmented.

Classifier	Hyperparameter	Value
Linear discriminant analysis	Hyperparameters optimization	Randomized GridSearch 5-Fold
	Solver	[“svd”, “lsqr”]
Logit	Hyperparameters optimization	Randomized GridSearch 5-Fold
	Penalty	[“l1”, “l2”, “elasticnet”, None]
	C	[1e-4, …, 1e4]
	Solver	[“liblinear”, “lbfgs”, “saga”, “newton-cg”]
	Max Iter	[100, 200, 500, 1,000]
	Class Weight	[None, “balanced”]
	Fit Intercept	[True, False]
Naive bayes	Hyperparameters optimization	Randomized GridSearch 5 Folds
	Var_smoothing	[1e-9,…, 1e-1]
Random forest	Hyperparameters optimization	GridSearch 5 Folds
	Number Of Estimators	[100, 200, 300, 500, 1,000]
	Maximum Depth	[None, 10, 20, 30, 50]
	Minimum Samples Split	[2, 5, 10]
	Minimum Samples Leaf	[1, 2, 4]
	Criterion	[“gini”, “entropy”, “log_loss”]
	Maximum Features	[“sqrt”, “log2”, None]
	Bootstrap	[True, False]
	Class Weight	[None, “balanced”]
Gradient boosting	Hyperparameters optimization	Randomized GridSearch 5 Folds
	Number Of Estimators	[100, 200, 300, 500, 1,000]
	Maximum Depth	[3, 4, 5, 10]
	Learning Rate	[0.01, 0.1, 0.2]
	Subsample	[0.5, 1.0]
	Column Sample by Tree	[0.8, 1.0]
	Minimum Samples Split	[2, 5, 10]
	Minimum Samples Leaf	[1, 2, 4]
	Maximum Features	[“sqrt”, “log2”, None]
	Loss	[“log_loss”, “exponential”]
Multi-layer perceptron with genetic Algorithm optimization.	Hyperparameters optimization	Genetic Algorithm
	Population Size	30
	Number of Generations	10
	Mutation Probability	0.2
	Crossover Probability	0.8
	Hidden Layer Sizes	[64,…, 623] or [8,…, 15]
	Dropout Rate	[0.1, 0.5]
	Learning Rate	[1e-5, 1e-3]
	Optimizer	Adam
	Loss	Binary Crossentropy
	Max Epochs	200
Multi-layer perceptron with Bayesian optimization	Hyperparameters optimization	Bayesian Optimization
	Hidden Layer Sizes	[64, …, 623] or [8, …, 15]
	L2 regularization	[0.0001, 0.01]
	Dropout Rate	[0.1, 0.5]
	Learning Rate	[1e-5, 1e-3]
	Optimizer	Adam
	Loss	Binary Crossentropy
	Max Epochs	200

For MLPs, optimization was performed using Bayesian Optimization (B) and Genetic Algorithms (GA), exploring configurations for layer size, dropout rate, learning rate, and regularization strength. In particular, GA-optimized ANNs have shown promising results in the literature and have been effectively used in related applications (34). Additionally, soft- and hard-voting classification strategies were also implemented to obtain two additional ensembled classifiers. GA-optimized MLP hyperparameters were set up with a population of 30 candidate models evolved over 10 generations, using a mutation probability of 0.2 and a crossover probability of 0.8 to explore alternative architectures and training settings. Each individual encoded a MLP with two hidden Dense layers (ReLU), where the first hidden layer size ranged from 64 to 623 units and the second from 8 to 15 units. Two dropout rates (one after each hidden layer) were sampled in the range 0.1–0.5 to mitigate overfitting, and the Adam learning rate was optimized within 1 × 10^−5^–1 × 10^−3^. Model fitness was assessed via 5-fold stratified cross-validation, using early stopping (patience = 10) during training (up to 200 epochs, batch size = 32). The GA-selected optimal hyperparameters were subsequently fixed and used to re-train the final MLP. The model was re-fitted under 5-fold stratified cross-validation (up to 200 epochs, batch size 32) with early stopping (patience = 10) and learning-rate scheduling (*ReduceLROnPlateau*). The B-optimized MLP was structurally similar, aiming for a more sample-efficient and guided exploration of the hyperparameters. Hidden layer sizes varied within the same ranges as GA, but in this setup the regularization scheme was richer, with an L2 penalty explicitly tuned between 0.0001 and 0.01, in addition to dropout between 0.1 and 0.5. As in evolutionary optimization, the learning rate was explored within the same interval, with Adam as optimizer, binary cross-entropy loss, and a maximum of 200 training epochs.

For each model, performance was evaluated using the Area Under the Receiver Operating Characteristic curve (AUC), Area Under the Precision-Recall curve (AUPRC), Accuracy, Sensitivity, and Specificity for both the discovery and validation sets. For OS status, deceased subjects were labelled as positives, and subjects alive at last follow-up as negatives. For recurrence, subjects experiencing recurrence were labelled as positives and those without recurrence as negatives.

### 2.5 MLP models explainability and clinical application

To compare the radiomics- and clinical-based models, a MLP classifier with one hidden layer of two neurons was implemented, taking into account established prognostic determinants for CRLM, including the Fong score, a prognostic index for recurrence after liver resection (11), the presence of bilateral disease, and a prior neoadjuvant chemotherapy; this approach facilitated the direct comparison of models across 5 folds in both the discovery and validation sets using a Wilcoxon signed-rank test.

Then, the SHAP algorithm (40) for eXplainable Artificial Intelligence was applied to the top-performing MLP classifiers, allowing the identification of the most influential radiomic features in the classification task.

Specifically, for each selected model, the top twenty features ranked by SHAP values from the discovery set were retained. Their distributions were dichotomized in the discovery set according to cutoffs determined by maximally selected rank statistics, generating two groups (“high” and “low”) using the surv_cutpoint function from the survminer R package (version 0.5.0), as previously described (41, 42). Each feature was then evaluated in the validation set using a univariate Cox proportional hazards model, and only those discriminating clinical outcomes with a *p*-value < 0.1 were retained. *P*-values were derived from pairwise comparisons using the z-statistic. The final set of radiomic features was further evaluated using the Kaplan–Meier (K–M) method. All survival analyses were conducted with the survival R package (version 3.6.4).

## 3 Results

### 3.1 Patients’ cohorts

As shown in Table 2, the discovery cohort included 197 patients, while the validation cohort comprised 34 patients. Progression or recurrence occurred in 66.5% and 76.5% of patients, respectively (*p*-value = 0.341). The OS status showed identical results in both cohorts (54.3% vs. 50.0%, *p*-value = 0.78), indicating a remarkably similar distribution between the two groups. Conversely, the median times of recurrence and OS were 22.33 months (Interquartile range [IQR]: 34.40–96.87) and 66.10 months (IQR: 34.40–96.87) in the discovery set and 13.47 months (IQR: 4.72–32.53) and 46.05 months (IQR: 33.75–62.90) in the validation set, respectively, with statistically significant difference (*p*-values = 0.010 and 0.017). Among all baseline characteristics, the most significant difference between the discovery and validation cohorts was observed for the distribution of the Fong clinical risk score (11), with 51 and 17 patients classified as high risks (3, 4, and 5 scores) from the discovery and validation sets (*p*-value = 0.008), respectively.

**Table 2:** Patients' characteristics for discovery and validation sets. Comparison of clinical determinant performance across discovery and validation sets using Wilcoxon signed-rank test and Fisher's exact test for continuous and categorical variables, respectively.

Variable	Discovery set	Validation set	*p*-value
*n*	197	34	
Progression or Recurrence = 1 (%)	131 (66.5)	26 (76.5)	0.341
OS status = 1 (%)	107 (54.3)	17 (50.0)	0.780
Local Progression or Recurrence = 1 (%)	81 (41.1)	18 (52.9)	0.272
OS (median [IQR])	66.1 [34.4, 96.9]	46.05 [33.8, 62.9]	0.017
Recurrence (median [IQR])	22.3 [9.7, 69.3]	13.37 [4.7, 32.5]	0.010
Age at resection (median [IQR])	61.0 [52.0, 69.0]	64.00 [56.3, 70.8]	0.109
Sex = F (%)	80 (40.6)	11 (32.4)	0.472
Multiple metastasis = Yes (%)	114 (57.9)	15 (44.1)	0.192
Fong score (%)			0.001
NA	29 (14.7)	0 (0.0)	
0	11 (5.6)	1 (2.9)	
1	42 (21.3)	6 (17.6)	
2	64 (32.5)	10 (29.4)	
3	44 (22.3)	10 (29.4)	
4	6 (3.0)	7 (20.6)	
5	1 (0.5)	0 (0.0)	
Fong score 3-4-5 vs. 0-1-2 = 3-4-5 (%)	51 (25.9)	17 (50.0)	0.008
CEA (median [IQR])	5.30 [2.3, 12.0]	8.70 [3.4, 39.5]	0.099
Max diameter (cm) (median [IQR])	2.80 [2.0, 4.3]	2.40 [2.0, 5.0]	0.873
Bilateral disease = Yes (%)	86 (43.7)	19 (55.9)	0.256
Previous neoadjuvant chemo = Yes (%)	122 (61.9)	15 (44.1)	0.078

OS, overall survival; IQR, interquartile range; CEA, carcinoembryoic antigen.

### 3.2 Classification results

As described in Section 3.1.1, the SMOTE algorithm was applied to the discovery set to balance clinical outcomes, yielding 262 observations for recurrence (131 events vs. 131 non-events) and 214 observations for OS status (107 events vs. 107 non-events).

**Figure 3:**
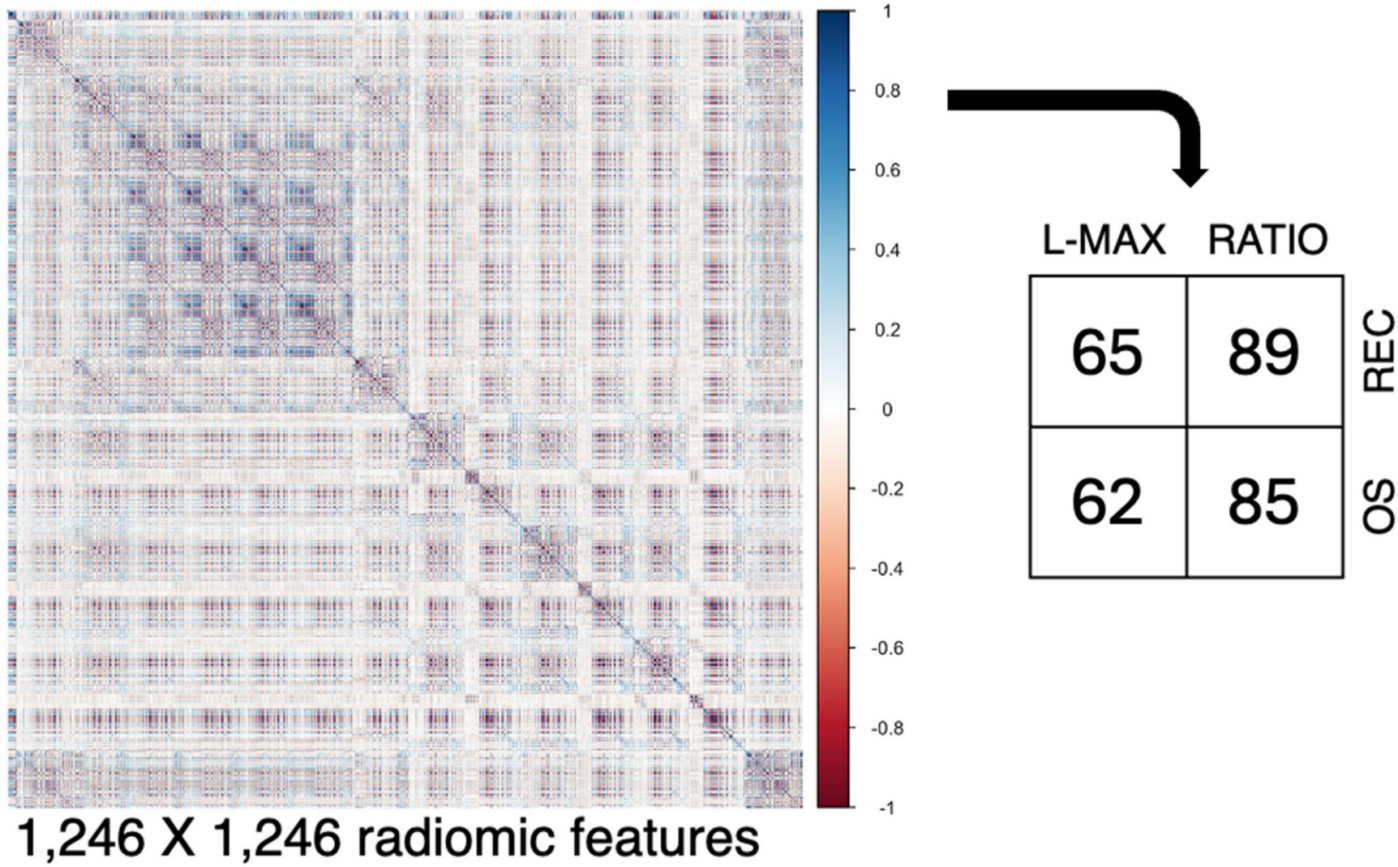
Collinearity analysis. From the segmented regions, 1,246 radiomic features were extracted, and their pairwise relationships were visualized using a Spearman correlation matrix. Abbreviations. REC, recurrence; OS, Overall Survival.

After PyRadiomics, 1,246 radiomic features were extracted (Figure 3). Pairwise correlations in a Spearman correlation matrix allowed us to select n. 65 and 62 features for L-MAX and n. 89 and 85 features for RATIO according to recurrence and OS status, respectively.

In consideration of the targets, a total of 48 distinct ML models were trained. This encompassed two sets of radiomic features (L-MAX and RATIO), including both the complete set and the reduced one, five traditional ML algorithms (LDA, Logit, NB, RF, and GB), and the TabNet classifier. In parallel, 24 models for the MLP classifiers were developed, utilizing both B and GA optimization strategies.

**Figure 4:**
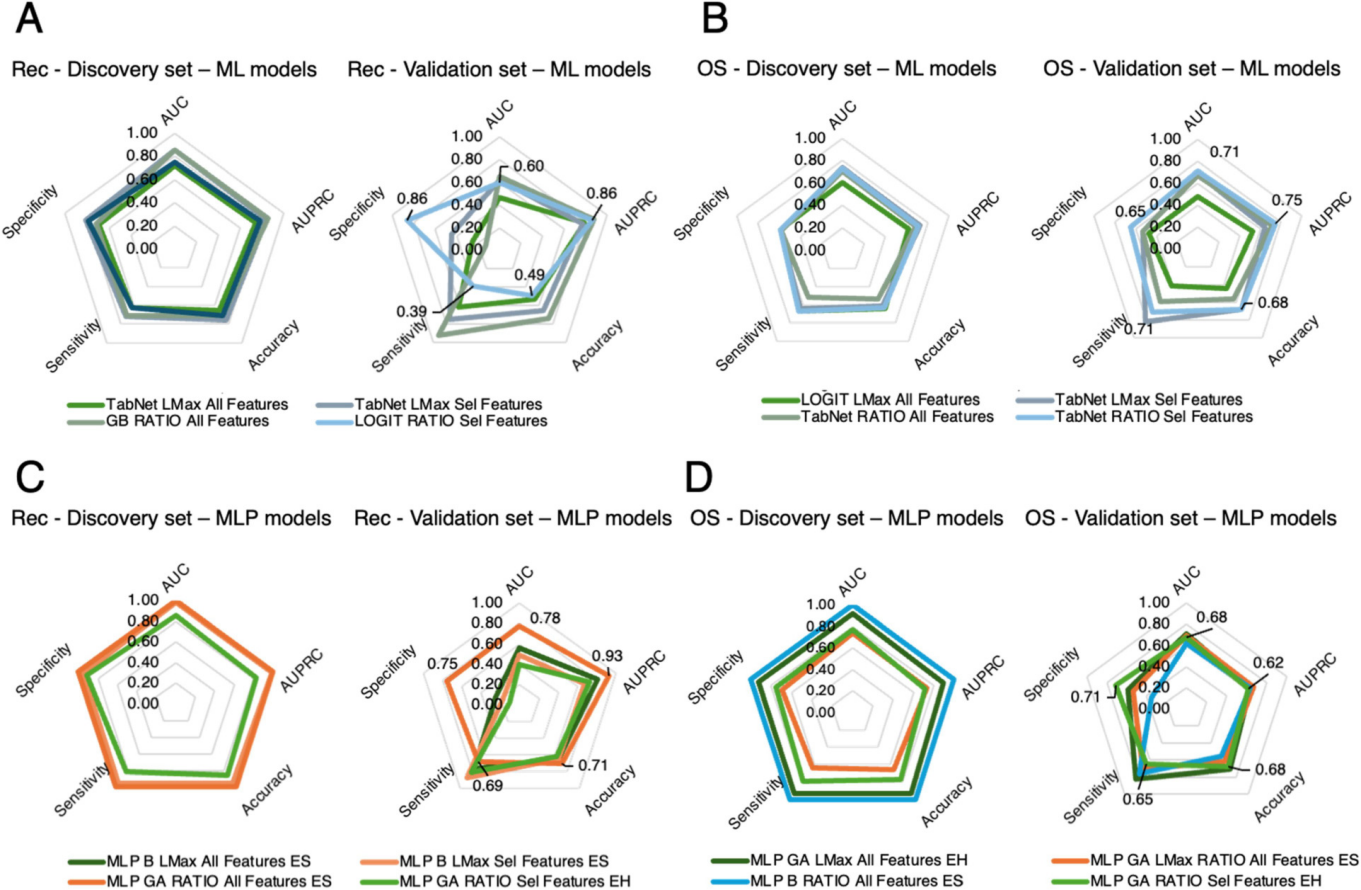
Radar plots comparing classification model performance across evaluation metrics for both the discovery set (5-fold cross-validation) and the external validation set. Plots are shown separately for recurrence prediction **(A,C)** and OS status prediction **(B,D)**. Colored outlines correspond to different classifiers or ensemble methods, while concentric pentagons represent metric scales from 0 (center) to 1 (outer edge). This visualization highlights trade-offs among metrics (e.g., sensitivity vs. specificity) and identifies models with the most balanced and robust performance across both datasets. Rec: Progression or Recurrence; ML: Machine Learning; OS: Overall Survival; GB: Gradient Boosting; Sel: Selected; AUC: Area Under Curve; AUPRC: Area Under Precision Recall Curve; MLP GA: Multi-Layer Perceptron Genetic Algorithm.

For recurrence, among the ML methods, the best performance was obtained with Logit for all features from RATIO distributions, achieving an AUC of 0.75 (AUPRC 0.78, accuracy 0.71, sensitivity 0.63, specificity 0.79) in the discovery set and 0.60 (AUPRC 0.86, accuracy 0.49, sensitivity 0.39, specificity 0.86) in the validation set (Figure 4A). Among the MLP-based approaches, the top-performing model was the configuration optimized with the GA, in a soft voting ensemble for all features from RATIO distributions, yielding an AUC of 1.00 (AUPRC 1.00, accuracy 1.00, sensitivity 1.00, specificity 1.00) in the discovery set and 0.78 (AUPRC 0.93, accuracy 0.71, sensitivity 0.69, specificity 0.75) in the validation set (Figure 4C).

For OS, the best ML model was TabNet for selected features from RATIO distributions, with an AUC of 0.73 (AUPRC 0.71, accuracy 0.63, sensitivity 0.67, specificity 0.59) in the discovery set and 0.71 (AUPRC 0.75, accuracy 0.68, sensitivity 0.71, specificity 0.65) in the validation set (Figure 4B). The best MLP-based approach was the GA-tuned MLP in a hard voting ensemble from the reduced RATIO distributions, achieving an AUC of 0.77 (AUPRC 0.71, accuracy 0.77, sensitivity 0.79, specificity 0.75) in the discovery set and 0.68 (AUPRC 0.62, accuracy 0.68, sensitivity 0.65, specificity 0.71) in the validation set (Figure 4D).

For comparison with clinical models, as depicted in Table 3A, for the lesion-only radiomic model (L-MAX), we employed for recurrence the Bayesian-optimized MLP, and for OS status the GA-optimized MLP, using all features in both cases. For recurrence, the clinical model showed modest and stable performance (AUC: 0.54 [95% confidence interval, CI: 0.45–0.63] in the discovery set and 0.49 [CI: 0.41–0.58] in validation; AUPRC: 0.54 [CI: 0.46–0.61] in the discovery set and 0.80 [CI: 0.75–0.84] in validation), whereas the radiomic model achieved near-perfect performance in the discovery set but exhibited a pronounced drop in validation (AUC: 0.46 [CI: 0.31–0.62]; AUPRC: 0.74 [CI: 0.67–0.81]; accuracy: 0.33 [CI: 0.09–0.58]). For OS status, the clinical model demonstrated low and variable discrimination (AUC: 0.55 [CI: 0.48–0.63] in discovery and 0.55 [CI: 0.39–0.71] in validation), while the radiomic model showed good performance in the discovery set (AUC: 0.86 [CI: 0.81–0.91]; AUPRC: 0.87 [CI: 0.81–0.94]) and moderate performance in validation (AUC: 0.55 [CI: 0.39–0.71]; AUPRC: 0.61 [CI: 0.48–0.74]; accuracy: 0.55 [CI: 0.47–0.63]).

**Table 3:** Performance of lesion-only (L-MAX) and lesion-on-background (RATIO) multilayer perceptron models for predicting recurrence (Rec) and overall survival (OS) in discovery (D) and validation (V) sets, showing that models including lesion-based features (A) and lesion-based features alongside with liver parenchymal background (B) consistently improve AUC, AUPRC, accuracy, sensitivity, and specificity compared with clinical-only models.

Target	Model	Set	AUC		AUPRC		Accuracy		Sensitivity		Specificity	
			mean [CI]	*p*-val	mean [CI]	*p*-val	mean [CI]	*p*-val	mean [CI]	*p*-val	mean [CI]	*p*-val
A—Lesion-only model, L-MAX												
Rec	MLP GA All Clin Feat	D	0.54 [0.45, 0.63]	0.01	0.54 [0.48, 0.61]	0.01	0.51 [0.49, 0.54]	0.01	0.66 [0.23, 1.10]	0.03	0.37 [−0.10, 0.84]	0.02
	MLP B L-MAX All Feat		0.99 [0.95, 1.03]		0.98 [0.94, 1.03]		0.98 [0.91, 1.04]		0.98 [0.92, 1.04]		0.97 [0.88, 1.05]	
	MLP GA All Clin Feat	V	0.50 [0.41, 0.58]	0.60	0.80 [0.75, 0.84]	0.17	0.62 [0.38, 0.85]	0.04	0.69 [0.30, 1.10]	0.04	0.38 [−0.01, 0.77]	0.01
	MLP B L-MAX All Feat		0.46 [0.31, 0.62]		0.74 [0.67, 0.81]		0.33 [0.09, 0.58]		0.25 [−0.10, 0.67]		0.93 [0.85, 1.01]	
OS status	MLP B All Clin Feat	D	0.55 [0.48, 0.63]	0.01	0.54 [0.48, 0.60]	0.01	0.54 [0.47, 0.62]	0.01	0.38 [−0.05, 0.81]	0.10	0.72 [0.32, 1.13]	1.00
	MLP GA L-MAX All Feat		0.86 [0.81, 0.91]		0.87 [0.81, 0.93]		0.78 [0.72, 0.83]		0.75 [0.59, 0.90]		0.80 [0.67, 0.94]	
	MLP B All Clin Feat	V	0.46 [0.35, 0.56]	0.29	0.59 [0.53, 0.64]	0.91	0.44 [0.30, 0.58]	0.21	0.42 [−0.04, 0.88]	0.40	0.56 [0.02, 1.11]	1.00
	MLP GA L-MAX All Feat		0.55 [0.39, 0.71]		0.61 [0.48, 0.74]		0.55 [0.47, 0.63]		0.59 [0.29, 0.89]		0.50 [0.16, 0.84]	
B—Lesion on Background model, RATIO												
Rec	MLP GA All Clin Feat	D	0.54 [0.45, 0.63]	0.01	0.54 [0.48, 0.61]	0.01	0.51 [0.49, 0.54]	0.01	0.66 [0.23, 1.10]	0.09	0.37 [−0.10, 0.84]	0.01
	MLP GA RATIO All Feat		0.99 [0.99, 0.99]		0.10 [0.99, 0.99]		0.97 [0.98, 1.00]		0.97 [0.94, 1.00]		0.97 [0.95, 1.00]	
	MLP GA All Clin Feat	V	0.49 [0.41, 0.58]	0.04	0.80 [0.75, 0.84]	0.21	0.62 [0.38, 0.85]	1.00	0.69 [0.29, 1.08]	0.69	0.38 [−0.01, 0.77]	0.83
	MLP GA RATIO All Feat		0.62 [0.54, 0.71]		0.83 [0.78, 0.87]		0.64 [0.54, 0.74]		0.69 [0.51, 0.86]		0.50 [0.23, 0.77]	
OS status	MLP B All Clin Feat	D	0.55 [0.48, 0.63]	0.01	0.54 [0.48, 0.60]	0.01	0.54 [0.47, 0.62]	0.02	0.38 [−0.05, 0.81]	0.15	0.72 [0.32, 1.13]	0.55
	MLP GA RATIO Sel Feat		0.74 [0.67, 0.81]		0.74 [0.67, 0.81]		0.68 [0.61, 0.76]		0.71 [0.57, 0.84]		0.65 [0.43, 0.88]	
	MLP B All Clin Feat	V	0.46 [0.35, 0.56]	0.02	0.59 [0.53, 0.64]	0.60	0.44 [0.30, 0.58]	0.08	0.42 [−0.04, 0.88]	0.29	0.56 [0.02, 1.11]	0.83
	MLP GA RATIO Sel Feat		0.61 [0.53, 0.70]		0.62 [0.54, 0.70]		0.60 [0.58, 0.61]		0.69 [0.44, 0.95]		0.49 [0.25, 0.74]	

AUC, area under the receiver operating characteristic curve; AUPRC, area under the precision–recall curve; CI, confidence interval; MLP, multilayer perceptron; GA, genetic algorithm; B, Bayesian; Clin, clinical; Feat, features.

On the other hand, for the lesion and background radiomic model (RATIO), as in Table 3B, we employed the GA-optimized MLP using all features for recurrence, and the GA-optimized MLP using selected features for OS status. For recurrence, the clinical model showed modest and stable performance (AUC: 0.54 [CI: 0.45–0.63] in the discovery set and 0.49 [CI: 0.41–0.58] in validation; AUPRC: 0.54 [CI: 0.46–0.61] in the discovery set and 0.80 [CI: 0.75–0.84] in validation), whereas the radiomic RATIO model achieved near-perfect performance in the discovery set (AUC: 0.99 [CI: 0.99–0.99]; AUPRC: 0.99 [CI: 0.99–0.99]) but exhibited reduced performance in validation (AUC: 0.62 [CI: 0.54–0.71]; AUPRC: 0.83 [CI: 0.78–0.87]; accuracy: 0.64 [CI: 0.54–0.74]). For OS status, the clinical model demonstrated low and variable discrimination (AUC: 0.55 [CI: 0.48–0.63] in discovery and 0.46 [CI: 0.35–0.56] in validation), while the radiomic RATIO model showed moderate performance in the discovery set (AUC: 0.74 [CI: 0.68–0.81]; AUPRC: 0.74 [CI: 0.67–0.81]) and retained comparable performance in validation (AUC: 0.61 [CI: 0.53–0.70]; AUPRC: 0.62 [CI: 0.54–0.70]; accuracy: 0.60 [CI: 0.58–0.61]).

**Figure 5:**
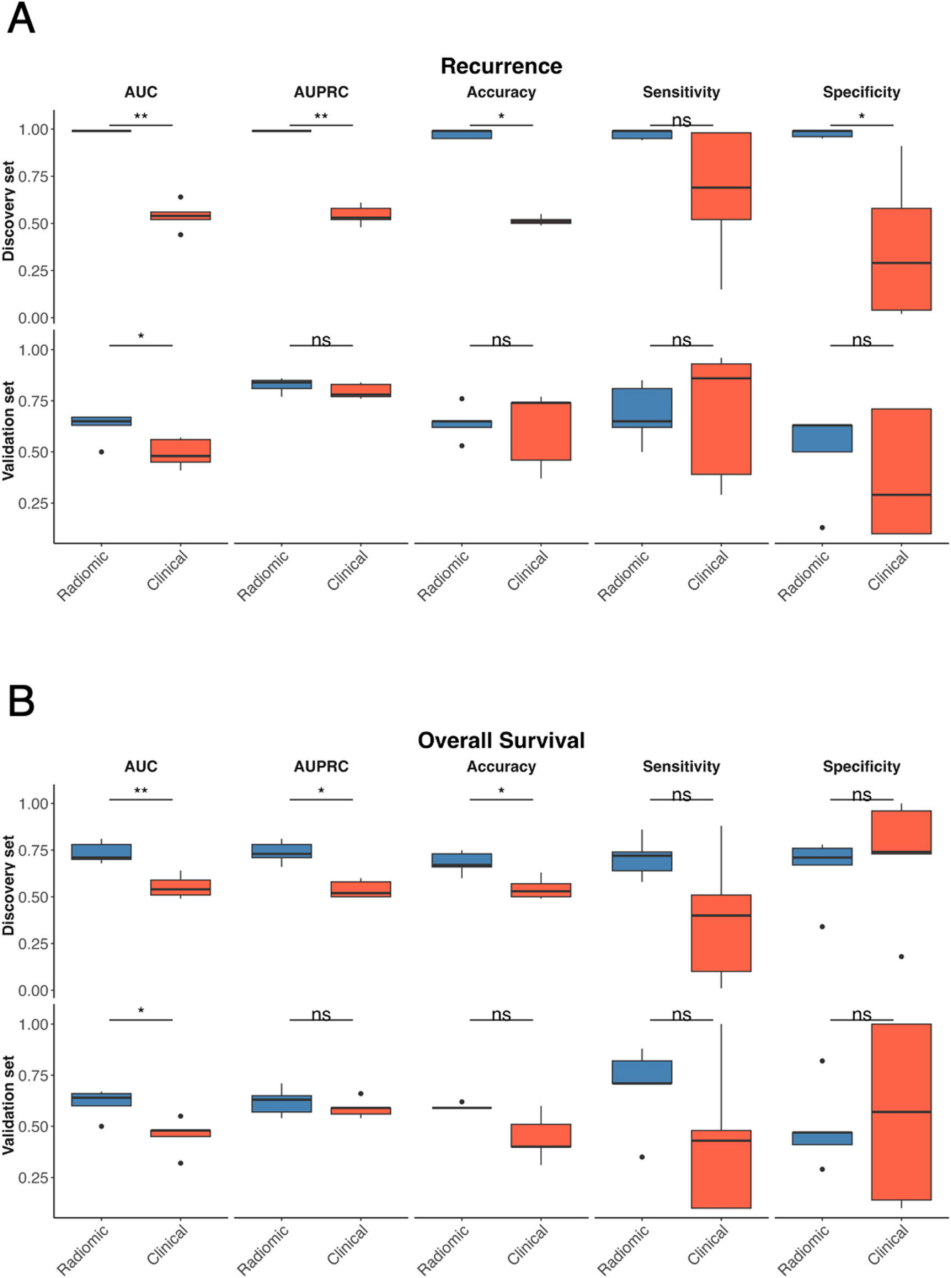
Comparison between radiomics and clinical MLP-based models. Comparison of radiomic (blue) and clinical (red) models for recurrence prediction across multiple performance metrics in the discovery and validation sets for recurrence **(A)** and OS status **(B)** Boxplots display the distribution of AUC, AUPRC, accuracy, sensitivity, and specificity values obtained from the 5-fold cross-validation procedure for the discovery set (top) and external validation set (bottom). Statistical comparisons were performed using the Wilcoxon singed-rank test, with significance levels indicated as ns (not significant), *p < 0.05, and **p < 0.01.

As shown in both Table 3B and Figure 5A, for recurrence prediction, radiomic models significantly outperformed clinical models in the discovery set for AUC (*p*-value = 0.01), AUPRC (*p*-value = 0.01), accuracy (*p*-value = 0.01), and specificity (*p*-value = 0.01), with no significant difference in sensitivity (*p*-value = 0.09); in the validation set, radiomic models retained a significantly higher AUC (*p*-value = 0.04), while all other metrics showed no significant difference. For OS status (Table 3B and Figure 5B), radiomic models significantly outperformed clinical models in the discovery set for AUC (*p*-value = 0.01), AUPRC (*p*-value = 0.01), and accuracy (*p*-value = 0.02), with no significant differences in sensitivity (*p*-value = 0.15) or specificity (*p*-value = 0.55); in the validation set, radiomic models again achieved a significantly higher AUC (*p*-value = 0.02), with all other metrics showing no significant differences.

### 3.3 Explainability analysis and clinical application

**Figure 6:**
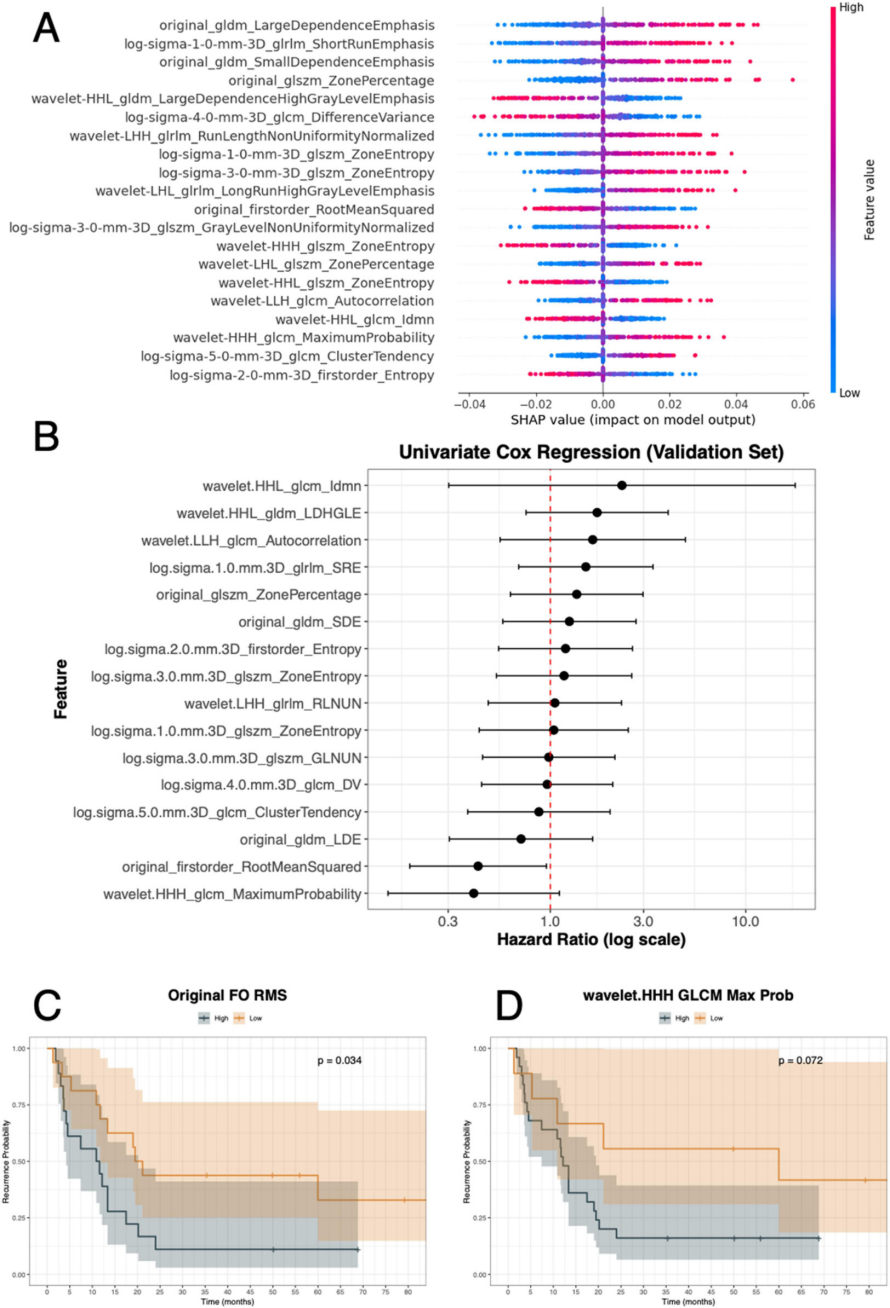
Recurrence analysis integrating model explainability and survival evaluation. **(A)** SHAP summary plot showing the top radiomic features contributing to recurrence prediction, with feature values (low to high) colored along the blue–red scale and SHAP values ranging approximately from −0.04 to +0.06. **(B)** Univariate Cox regression analysis of selected features in the validation set, displaying hazard ratios with 95% confidence intervals. **(C,D)** Kaplan–Meier survival curves for two representative features: Original first-order RootMeanSquared (*p*-value = 0.034) and wavelet.HHH_glcm_MaximumProbability (*p*-value = 0.072), stratified into high vs. low groups. gldm: Gray Level Dependence Matrix; glrlm: Gray Level Run Length Matrix; glszm: Grey Level Size Zone Matrix; glcm or GLCM: Gray Level Co-Occurrence Matrix; FO: First Order; RMS: Root Mean Squared; Prob: probability.

The SHAP summary plot showing the top 20 radiomic features contributing to recurrence prediction with the GA-optimized MLP in a soft-voting ensemble using all RATIO-based features is depicted in Figure 6A. Each point represents a patient, with colours indicating feature values (blue = low, red = high) and position on the *x*-axis showing the impact on the model output. For the discovery set, SHAP values ranged approximately from −0.04 to +0.06, where positive values increased the predicted risk of recurrence and negative values decreased it. For instance, higher values of features such as original_GLDM_LargeDependenceEmphasis, log-sigma-1-0-mm-3D_glrlm_ShortRunEmphasis, and original_GLDM_SmallDependenceEmphasis were associated with an increased recurrence risk. After evaluating each feature in a univariate Cox model from the validation set, two radiomic features reached significance or borderline significance (Figure 6B and Table 4). In fact, original_firstorder_RootMeanSquared showed a significant protective effect (Hazard Ratio [HR] = 0.43, CI: 0.19–0.95, *p*-value = 0.04), with lower values associated with higher recurrence risk and wavelet.HHH_GLCM_MaximumProbability demonstrated a borderline association (HR = 0.41, 95% CI: 0.15–1.11, *p*-value = 0.08). Again, for the validation set, we assessed survival curves by K–M for these two radiomic features (Figures 6C,D). Consistently with SHAP and Cox, patients with high levels of original_firstorder_RootMeanSquared and wavelet.HHH_GLCM_MaximumProbability achieved higher probability of recurrence than those patients with low levels (*p*-values = 0.034 and 0.072).

**Table 4:** Univariate Cox regression analysis of radiomic features for recurrence and overall survival (OS) in the validation set, showing that most lesion-based texture and intensity descriptors are not significantly associated with outcomes.

Endpoint	Feature	HR	Lower CI	Upper CI	*p*-value
Recurrence	wavelet.HHL_glcm_Idmn	2.33	0.30	17.92	0.42
	wavelet.HHL_gldm_LDHGLE	1.74	0.75	4.01	0.20
	wavelet.LLH_glcm_Autocorrelation	1.65	0.55	4.91	0.37
	log.sigma.1.0.mm.3D_glrlm_SRE	1.52	0.69	3.35	0.30
	original_glszm_ZonePercentage	1.36	0.62	2.98	0.44
	original_gldm_SDE	1.25	0.57	2.75	0.57
	log.sigma.2.0.mm.3D_firstorder_Entropy	1.20	0.54	2.63	0.66
	log.sigma.3.0.mm.3D_glszm_ZoneEntropy	1.18	0.53	2.61	0.69
	wavelet.LHH_glrlm_RLNUN	1.05	0.48	2.31	0.89
	log.sigma.1.0.mm.3D_glszm_ZoneEntropy	1.04	0.43	2.51	0.93
	log.sigma.3.0.mm.3D_glszm_GLNUN	0.98	0.45	2.14	0.96
	log.sigma.4.0.mm.3D_glcm_DV	0.96	0.44	2.09	0.92
	log.sigma.5.0.mm.3D_glcm_ClusterTendency	0.87	0.38	2.02	0.75
	original_gldm_LDE	0.71	0.30	1.65	0.42
	**original_firstorder_RootMeanSquared**	**0**.**43**	**0**.**19**	**0**.**95**	**0**.**04**
	**wavelet.HHH_glcm_MaximumProbability**	**0**.**41**	**0**.**15**	**1**.**11**	**0**.**08**
OS	**wavelet.HLL_glszm_SALGLE**	**2**.**56**	**0**.**90**	**7**.**28**	**0**.**08**
	wavelet.HHL_firstorder_Skewness	1.62	0.45	5.75	0.46
	original_glcm_ClusterShade	1.52	0.55	4.19	0.42
	wavelet.HLH_glcm_ClusterShade	1.43	0.32	6.34	0.64
	wavelet.HHL_firstorder_Median	1.03	0.13	7.84	0.98
	wavelet.LLL_glszm_SALGLE	0.98	0.36	2.65	0.97
	wavelet.HHH_glcm_ClusterShade	0.90	0.20	4.01	0.89
	log.sigma.5.0.mm.3D_firstorder_90P	0.88	0.32	2.41	0.81
	wavelet.HHL_glcm_ClusterShade	0.80	0.29	2.19	0.67
	wavelet.HHH_firstorder_Skewness	0.72	0.23	2.21	0.57
	wavelet.LHL_glcm_ClusterShade	0.70	0.26	1.91	0.49
	wavelet.HHL_glcm_Imc1	0.67	0.15	3.00	0.60
	wavelet.HLL_glcm_ClusterShade	0.64	0.22	1.84	0.41
	wavelet.LHL_glszm_SAE	0.59	0.22	1.60	0.30
	wavelet.LHL_firstorder_Skewness	0.56	0.13	2.45	0.44
	wavelet.HHH_firstorder_Mean	0.50	0.06	3.95	0.51
	**log.sigma.1.0.mm.3D_glcm_ClusterShade**	**0**.**40**	**0**.**15**	**1**.**04**	**0**.**06**

HR, hazard ratio; CI, confidence interval; OS, overall survival.

Bold features are statistically significant or strictly close to the statistically significance.

**Figure 7:**
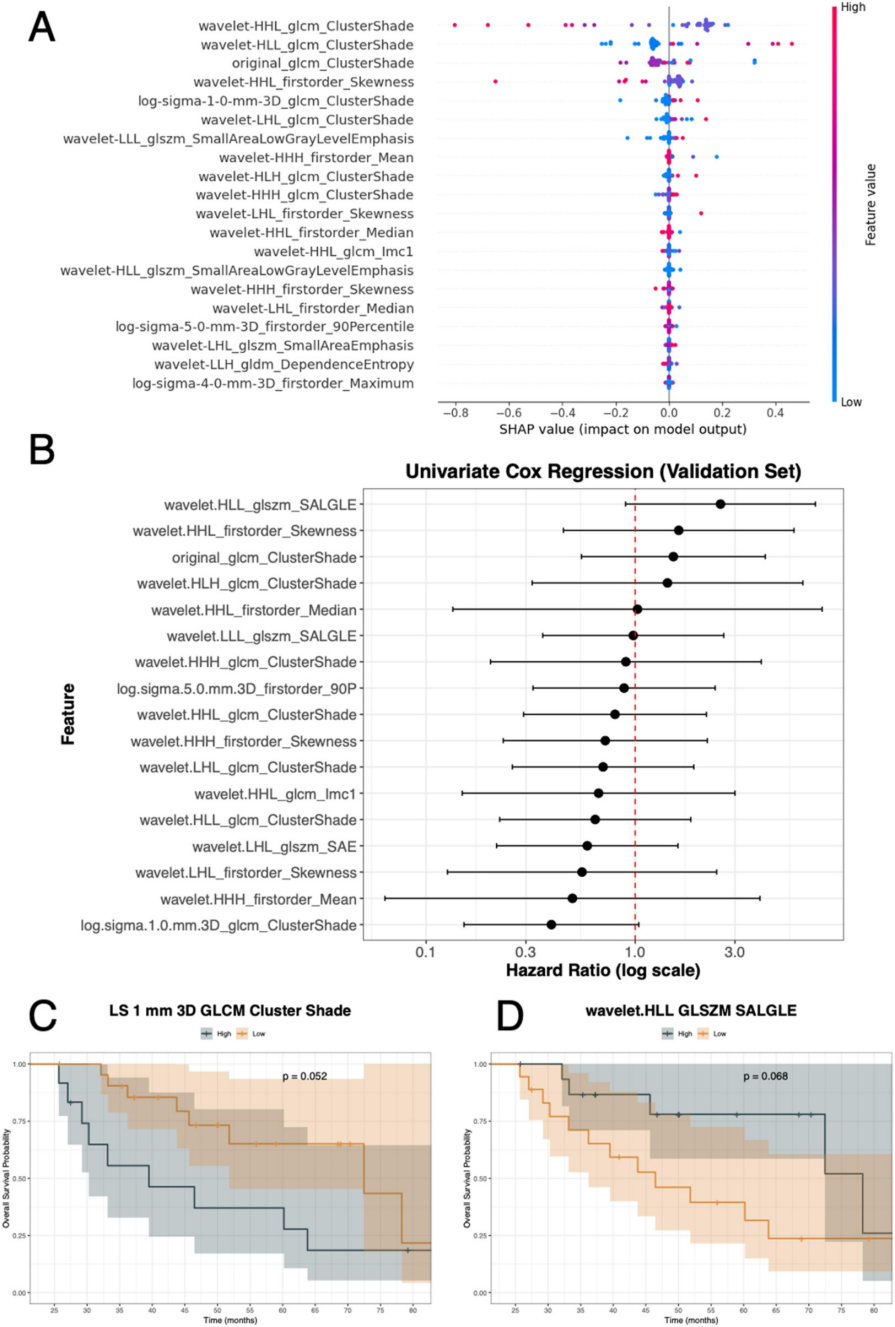
OS analysis integrating model explainability and survival evaluation. **(A)** SHAP summary plot displaying the top radiomic features contributing to OS status prediction, with feature values (low to high) shown along the blue–red scale and SHAP values ranging from approximately −0.8 to +0.4. **(B)** Univariate Cox regression analysis of selected features in the validation set reporting hazard ratios with 95% confidence intervals. **(C,D)** Kaplan–Meier survival curves for two representative features: log-sigma-1.0-mm-3D GLCM ClusterShade (*p*-value = 0.052) and wavelet.HLL_glszm_SALGLE (*p*-value = 0.068), stratified into high vs. low groups. glcm or GLCM: Gray Level Co-Occurrence Matrix; glszm: Grey Level Size Zone Matrix; gldm: Grey Level Dependence Matrix.

Figure 7 shows the clinical application according to the OS status. The SHAP summary plot depicts the top 20 radiomic features for OS status from the discovery set using the GA-optimized MLP in a hard voting ensemble using all RATIO-based features (Figure 7A). SHAP values ranged approximately from −0.8 to +0.4, where positive values increased the risk of death and negative values decreased it. Features such as wavelet-HHL_GLCM_ClusterShade, wavelet-HLL_GLCM_ClusterShade, original_GLCM_ClusterShade, and wavelet-HHL_firstorder_Skewness showed the strongest impact on OS status. From the validation set, univariate Cox analysis for OS, two radiomic features showed borderline significance (Figure 7B and Table 4), log.sigma.1.0.mm.3D_GLCM_ClusterShade was associated with a protective trend (HR = 0.40, 95% CI: 0.15–1.04, *p*-value = 0.06) and wavelet.HLL_GLSZM_SALGLE (Small Area Low Gray Level Emphasis) suggested an increased risk of death (HR = 2.56, 95% CI: 0.90–7.28, *p*-value = 0.08). Again, for the validation set, we assessed survival curves by K-M for these two radiomic features. Consistently with SHAP and Cox, patients with high levels of log.sigma.1.0.mm.3D_GLCM_ClusterShade (Figure 7C) achieved higher probability of death than those patients with low levels (*p*-value = 0.05). On the contrary, patients with low levels of wavelet.HLL_GLSZM_SALGLE (Figure 7D) achieved higher probability of death than those patients with high levels (*p*-value = 0.07).

## 4 Discussion

This study aimed to develop and validate explainable ML pipelines using radiomics to predict outcomes in patients with CRLM, focusing on both metastatic lesions and surrounding liver tissue. The objective was not only to achieve high prognostic performance, comparable with recognized clinical tools and externally validated in a real-life cohort, but also to provide interpretable models. The results showed that incorporating radiomic features from the largest lesion, as well as contextual information from background liver tissue, improved the prediction of both recurrence and OS status. The most accurate models were TabNet and GA-optimized MLPs, with SHAP explainability analysis identifying key imaging features driving model predictions. The use of relatively complex MLP architectures for high-dimensional data was motivated by the need to balance under- and overfitting by aligning model capacity with the underlying non-linear signal. GA optimisation was not used to arbitrarily increase complexity, but rather to select parsimonious MLP architectures and feature subsets that achieved stable performance under cross-validation and on the independent validation set, while still capturing non-linear patterns that simpler linear models, such as logistic regression, may miss. External validation confirmed the models' clinical applicability and robustness, marking an advancement in both predictive power and interpretability compared to traditional models. Survival modeling was incorporated at the explainability stage, thereby preserving a time-to-event interpretation of the results and ensuring clinical interpretability for both recurrence and OS. This approach allowed the study to combine the robustness and practicality of a binary classification framework for model training with a clinically meaningful temporal perspective when interpreting model outputs.

Recent studies have applied radiomics and ML for CRLM prognosis, with performance metrics ranging from moderate to strong accuracy. For example, Miyamoto et al. reported that a CT radiomics-driven Random Forest model for chemotherapy response yielded an AUC of 0.87 in the validation cohort (43). The explainable ML models developed in this study achieved AUC values up to 0.78 for recurrence prediction and 0.71 for OS status in the external validation cohort, with accuracy values reaching 0.71 and 0.68, respectively. The final classifiers selected were the GA-optimized MLP in a soft voting ensemble for recurrence and the GA-optimized MLP in a hard voting ensemble for OS status. These models integrated radiomic features from the largest lesion and background liver tissue, leveraging contextual ratios to enhance predictive power and clinical interpretability. These results demonstrate that considering tumor radiomic values alongside healthy parenchymal liver background representation provides a substantial and consistent quantitative gain in all main performance indicators in both the discovery and validation cohorts. This finding is in line with what was recently reported by Marzi et al. (29). Compared to state-of-the-art research, Fu et al. developed a CT-based radiomics model with an AUCs ranging among 0.72 and 0.93 in the external validation set for predicting group with DFS ≥12 months vs. recurrence group with DFS <12 months after neoadjuvant therapy in patients with CRLM (21). Granata et al. applied KNN models for liver recurrence detection from CT and MRI images, reporting AUCs between 0.80 and 0.97, and accuracy of 0.91 (23, 24). Mühlberg et al. constructed CT geometric and radiomic models achieving AUCs of 0.73 for OS prediction at 1-year in cross-validation (22). Thus, considering methodological diversities (e.g., the recurrence definition), the models in the present study demonstrate performance that is comparable to results available for CRLM outcome prediction in current literature.

Importantly, the present approach offers significant advances in interpretability and external validation, addressing key weaknesses of prior studies. The use of SHAP analysis enabled identification of key radiomic features contributing to prognosis, and external validation on a real-life cohort confirmed the models' robustness and clinical relevance. The most influential radiomic features for both recurrence and OS, as identified through SHAP analysis within the selected MLP ensembles, maintained their prognostic value in the independent validation cohort. For recurrence, features like original_firstorder_RootMeanSquared and wavelet.HHH_GLCM_MaximumProbability consistently contributed to model prediction and successful patient stratification. In the survival models, log.sigma.1.0.mm.3D_GLCM_ClusterShade and wavelet.HLL_GLSZM_SALGLE emerged as key determinants, reflecting distinct aspects of tissue heterogeneity and intensity distribution. These features, rooted in quantitative texture and intensity patterns, captured relevant biological and clinical information, supporting their use for risk assessment in CRLM. The external validation of these determinants demonstrates the robustness and generalizability of the radiomics-based approach, further underscoring its potential utility in personalized prognostic modelling. In a clinical setting, imaging-based prediction of recurrence and OS could help stratify patients into higher- and lower-risk groups, informing decisions on surveillance intensity, perioperative or adjuvant systemic treatments, and selection for more aggressive local or combined multimodal approaches. In addition, such risk stratification may support the identification of candidates for clinical trials specifically targeting patients with unfavourable imaging-derived risk profiles. Moreover, another strength of our work is the comprehensive validation strategy. We applied cross-validation and independent test splits to ensure model generalizability. Furthermore, we compared multiple classifiers to identify the best-performing architecture for this specific clinical application.

This study presents several limitations that should be considered when interpreting the results. The clinical cohorts included were slightly diverse (recurrence and OS median follow-up of the validation set are significantly shorter than the discovery set), which may introduce variability and affect the comparability of findings across patient populations. To address this, sample balancing in the discovery set was implemented to enhance generalizability, though this approach may have influenced feature selection and model outcomes. Additionally, not all radiomic variables identified by SHAP in the discovery phase retained prognostic value in the validation set, suggesting that feature stability can be context dependent. Some of these selected features did not consistently demonstrate significant associations with clinical outcomes, highlighting the need for continued refinement and external validation of radiomic markers for robust prognostic use.

## 5 Conclusions

In this work, we designed, developed, and externally validated ML-based classifiers for the prediction of the prognosis of CRLM patients in terms of recurrence and OS. The implemented approach, based on radiomic features extracted from PV phase CT, analysed both lesion-centric and contextual liver parenchyma information.

Furthermore, model transparency was emphasized through SHAP, which allowed the identification of biologically plausible determinants for establishing the prognostic outcome.

The obtained results demonstrated both the robustness and the applicability of the implemented approach; by combining lesion characteristics and parenchyma context, the optimized MLP models uncovered sub-visual, context-aware imaging patterns, not immediately appreciable with routine assessments, thereby improving prognostic stratification. Furthermore, analyses revealed that such an approach allowed overcoming established clinical scores, whose generalizability can be limited across heterogeneous clinical settings. Supporting decisions with transparent, reproducible radiomic determinants and validating automatic models on an independent cohort further supports consistent risk estimation across variable scanners and populations, paving the way for prospective clinical integration in decision support and trial enrichment.

The next phase of this research will focus on expanding the analytic framework and integrating additional data modalities to further refine prognostic modelling for CRLM. First, the application of survival explainability techniques will enable more nuanced interpretation of time-to-event outcomes, enhancing clinical utility. Advanced DL methods will be employed to extract higher-level image features, with the potential to uncover complex patterns not readily captured by traditional radiomics. Finally, radiomic predictors will be combined with biological and mutational attributes in a multi-omic approach, aiming to improve patient stratification and outcome prediction through a comprehensive analysis of imaging, molecular, and genetic data.

## Data Availability

The raw data supporting the conclusions of this article will be made available by the authors, without undue reservation.
